# A Rare Pediatric Case of Chronic Toe Osteomyelitis Due to Contiguous Spread

**DOI:** 10.7759/cureus.87778

**Published:** 2025-07-12

**Authors:** Elpis Chochliourou, Ioannis Trevlias, Maria Ziaka, Aikaterini Tzantzaroudi, Charalampos Antachopoulos

**Affiliations:** 1 School of Medicine, Aristotle University of Thessaloniki, Thessaloniki, GRC; 2 Third Department of Pediatrics, Infectious Diseases Unit, Hippokration General Hospital, Thessaloniki, GRC; 3 First Department of Pediatric Surgery, G. Gennimatas General Hospital, Thessaloniki, GRC

**Keywords:** child, contiguous spread, onychomycosis, toe amputation, toe osteomyelitis

## Abstract

Osteomyelitis is a relatively rare infection in children that can develop in the context of foot nail injuries. Contiguous osteomyelitis may develop from trauma, inoculation during surgical procedures, or from neighboring infected tissue. We present a rare case of a patient with contiguous osteomyelitis following surgical nail removal due to persistent onychomycosis. Considering the severity of the infection, a phalanx amputation was performed. Chronic osteomyelitis, which can result from the contiguous spread of infection following an injury, may lead to serious complications. Each type of osteomyelitis requires different treatment strategies, either medical or surgical. Prompt diagnosis is crucial for both prognosis and successful treatment. The latter mandates interdisciplinary interventions combining patient assessment, antibiotics, and surgery.

## Introduction

Osteomyelitis is defined as a bone infection related to a microbial factor. It is an infrequent complication of minor trauma, such as foot nail injuries, among the pediatric population. Chronic osteomyelitis is a severe condition characterized by bone necrosis [1,2]. Prompt diagnosis is crucial for the prognosis. We report a case of chronic osteomyelitis resulting from the contiguous spread of infection following a toe injury. A history of onychomycosis of the same toe further complicated the clinical outcome. The severity of infection finally resulted in amputation of the distal and medial phalanx of the patient’s right great toe.

## Case presentation

An otherwise healthy prepubescent boy presented to the pediatric surgery department of our institution complaining of deterioration of a previous injury to his right great toe, which was accompanied by severe pain.

**Figure 1:**
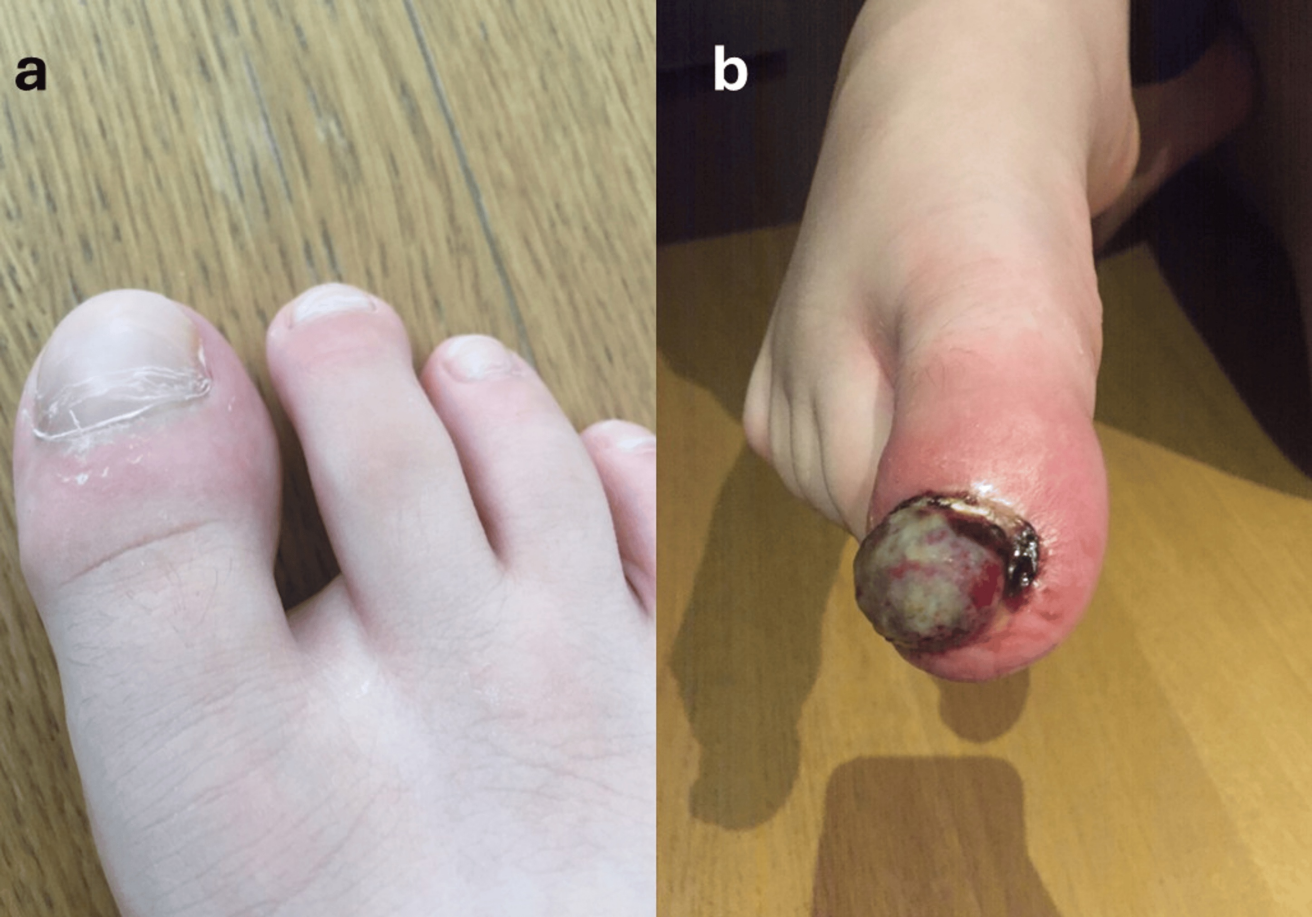
(a) Persistent onychomycosis of the nail before removal. (b) Persistence of regional inflammation and formation of granulomatous tissue after the toe injury.

The patient had undergone a nail removal three months before due to persistent onychomycosis (Figure 1a). Initially discharged on local antibiotics without success, the boy then received a 10-day course of oral cefprozil because of persisting inflammation. Three weeks after the excision, an accidental trauma in the area contributed to the formation of granulomatous tissue in the toe (Figure 1b). The patient underwent a second procedure for surgical debridement. After the second procedure, the patient was discharged on oral cefprozil.

On the day of presentation to the pediatric surgery department, the signs of lingering inflammation prompted a CT scan of the right foot, which revealed disturbance of regional architecture in the medial and distal phalanx of the great toe, loss of bone beams and cortical thinning with ruptures of the periosteum, accompanied by swelling of soft tissue.

**Figure 2:**
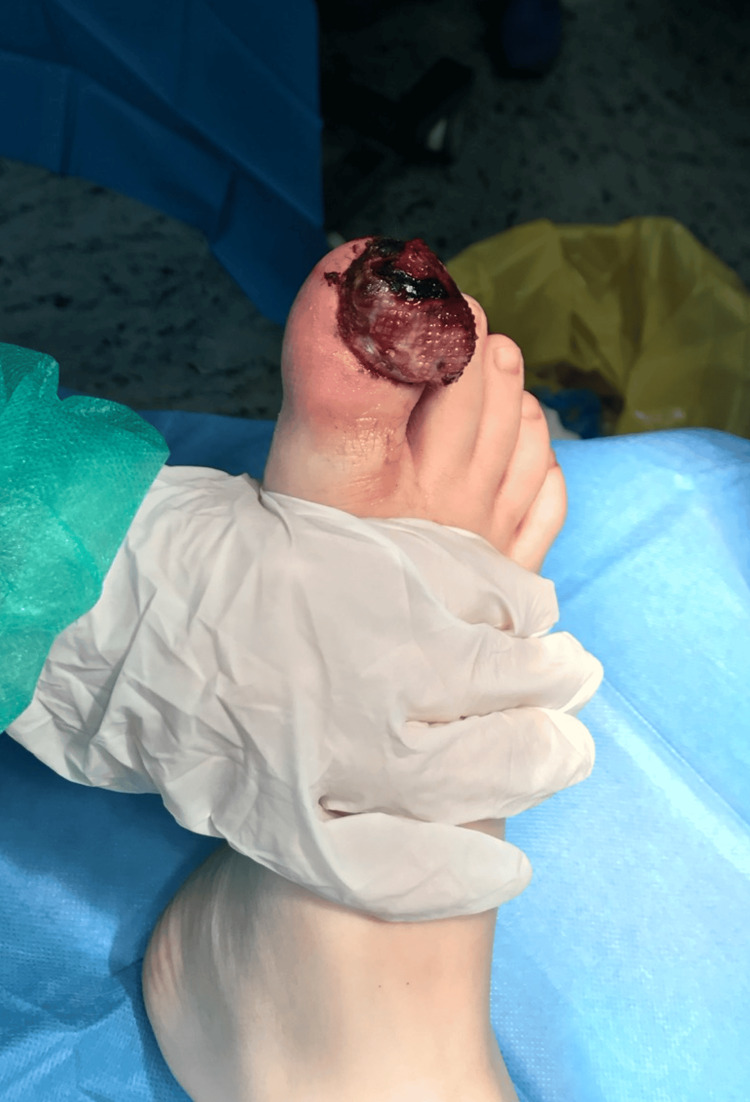
Intraoperative image showing the infected area with giant granular tissue and edema.

Due to the abovementioned signs, in addition to the remarkable depth of inflammation on imaging, osteomyelitis was suspected. The patient was started on a four-day antibiotic regimen with oral amoxicillin/clavulanate, and an additional surgical debridement was performed (Figure 2). The procedure revealed complete dissolution of the bone architecture of the distal phalanx of the involved digit, which was subsequently amputated. Bone specimens were sent for culture and submitted for biopsy.

Initial laboratory results revealed a white blood cell count of 4.3 (x10^9^/L), with neutrophil predominance (68.2%) and a CRP value of 12 mg/L, which returned to normal during hospitalization.

The child was subsequently referred to the department of pediatrics at our hospital for further therapeutic management. The patient was started on IV (intravenous) amoxicillin/clavulanate 1 g TID plus IV itraconazole, 250 mg BID. The antifungal was added due to the onychomycosis that led to the initial nail removal, as well as the absence of elevated inflammatory markers typically present in bacterial osteomyelitis. All blood cultures were negative for bacteria or fungi. Plain radiograph imaging was consistent with osteomyelitis. Direct microscopy of tissue biopsy specimens obtained from the excised phalanx revealed granular inflammation with neutrophil and giant-cell infiltration of the dermis. At the same time, periodic acid-Schiff and methenamine silver stains were negative for fungi. Tissue specimen cultures isolated methicillin-sensitive *Staphylococcus aureus* and *Enterococcus faecalis*, which were sensitive to the initial empiric regimen, without any fungal growth. Due to the rarity of the infection and its severity, which led to phalanx amputation, additional testing was performed to assess the integrity of the immune system function before discharge. The immunophenotypic profile of the peripheral blood was normal, while dihydrorhodamine flow cytometric testing revealed no abnormalities in phagocytic function. Serum immunoglobulin levels were not indicative of deficiencies in humoral immunity.

**Figure 3:**
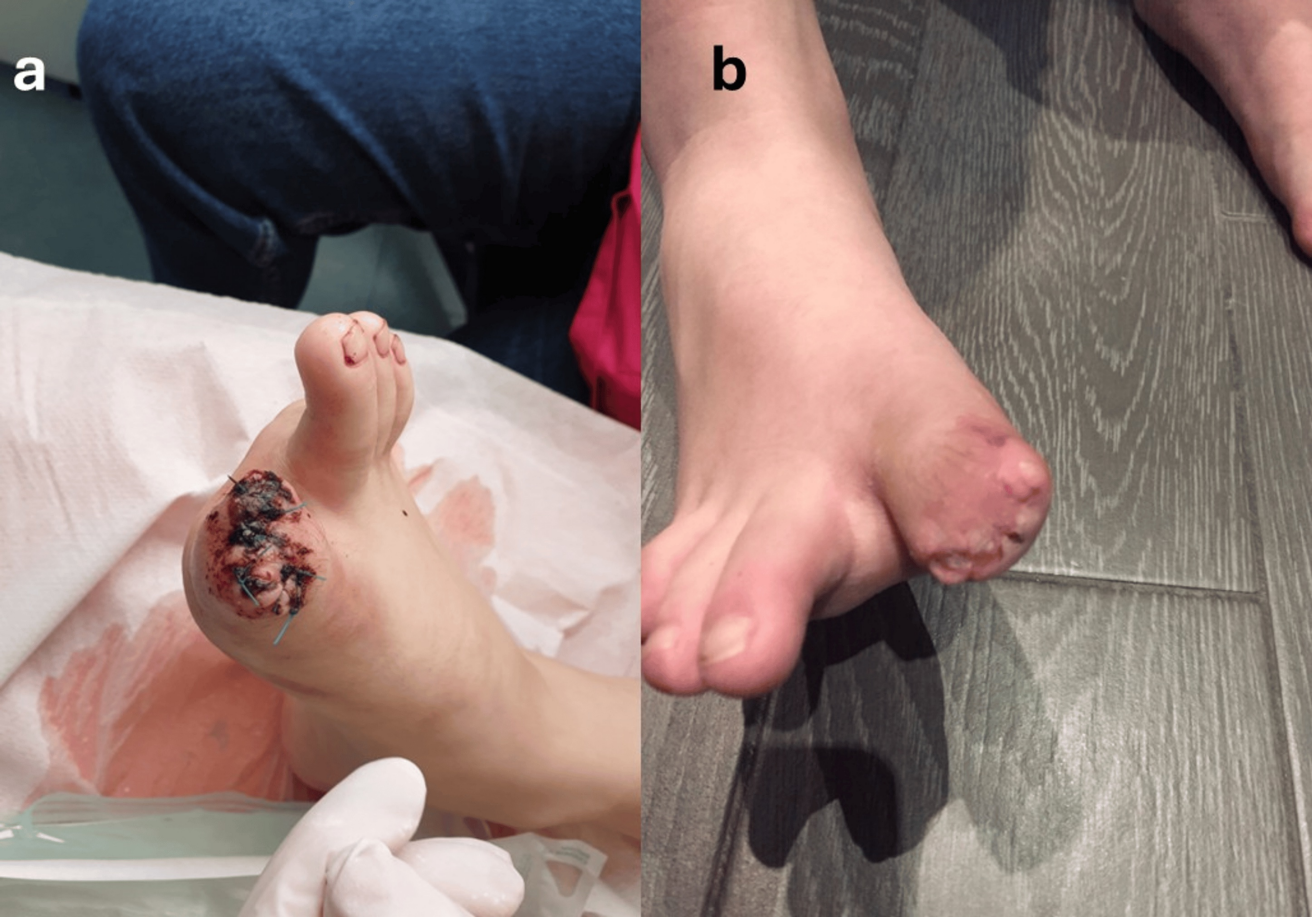
(a) Clinical image shortly after operation. (b) Absence of inflammation during the healing process postoperatively.

During the entire hospitalization in our institution, the boy remained afebrile and in good general condition. He was discharged after nine days on oral amoxicillin/clavulanate, 500 mg TID for a month (Figure 3).

Currently, seven months after the phalangeal amputation, the patient remains in excellent condition and is entirely functional, with no clinical evidence of infection.

## Discussion

Overall, reported cases of osteomyelitis vary globally, ranging from up to 10 cases per 100,000 in high-income countries to as many as 80 cases per 100,000 in low-income countries [1]. Osteomyelitis is chiefly hematogenous and monomicrobial among pediatric patients. The disease, however, can spread to adjacent infected tissue through trauma or as a result of surgery [2,3]. The lack of substantial tissue between anatomical loci on the hands and feet of children is a probable factor that accounts for the easier contiguous spread of infection [2,4,5].

Protein-rich tissue (e.g., collagen) exposed due to trauma presents a readily available medium for microbes to bind to. Thus, bone fractures, whether open or closed, are more prone to infection [6,7]. Lack of medical resources in parts of the world leads to infectious complications in 44% of open fractures and 2-60% of puncture wounds [8-10]. Prolonged infection leads to the formation of necrotic bone lesions. This more severe, surgical condition is referred to as chronic osteomyelitis [11].

Clinical manifestations of osteomyelitis are heterogeneous. The physician must be highly suspicious of the disease for a favorable prognosis. A definitive diagnosis is made through bone tissue culture and biopsy, which also guides the selection of antibiotic treatment. The most common causal organism is *Staphylococcus aureus* (methicillin-sensitive or methicillin-resistant). Important pathogens also include *Streptococcus* Group B, *Escherichia coli*, *Pseudomonas aeruginosa*, and *Kingella kingae* [2,3].

Lab test results involving leukocyte counts and inflammatory markers are often not reliable. False negative culture results have been reported at a rate of up to 40%, attributed to difficulties in culturing and prior antibiotic administration. The late onset of radiologic imaging findings (up to three weeks in some cases) results in a low early diagnostic value, adding to the challenge. However, they are advised, particularly in the presence of cellulitis or an abscess of the extremities [6,12-14].

Our case had no specific evidence for microbial osteomyelitis. Combined with a history of persistent onychomycosis, these were the reasons for thinking a fungus rather than a microbe was the causative factor. Fungal infections of bone and soft tissue can occur in children post-injury. They can manifest serious clinical presentations, especially among the immunocompromised, and require long-term antifungal regimens [15].

Treatment for chronic osteomyelitis mandates multidisciplinary interventions. Apart from antibiotics (which are usually administered for extended periods), the cornerstone of treatment is surgical debridement of the necrotic bone tissue [16,17]. Debridement ought to be thorough, and material removed until the encounter of living, adequately vascularized tissue. Resection of inadequate amounts of tissue is related to recurrence [18,19].

Advances in clinical knowledge, new diagnostic methods, and earlier and more appropriate antibiotic therapy have led to a decrease in the rate of treatment failure concerning osteomyelitis in children. Surgical treatments involve a multitude of specialties to ensure a better functional and cosmetic result. Further research is investigating the role of growth factors for bone regeneration, with expected favorable results such as a swifter recovery and reduced vulnerability to infection [11].

## Conclusions

Osteomyelitis is a clinical entity that mandates several days of hospitalization. It is certainly not the first diagnosis that comes to the physician’s mind immediately after a superficial toe infection, but it should remain within the differential diagnosis. The contiguous spread of infection to adjacent bone tissue requires a multidisciplinary team of experts to mitigate it. This mitigation does not always entail a full recovery with intact extremities and may include amputating measures to ensure a positive outcome. Proper and timely treatment benefits both the patient and the hospital.

## References

[1] Congedi S, Minotti C, Giaquinto C, Da Dalt L, Donà D (2020). Acute infectious osteomyelitis in children: new treatment strategies for an old enemy. World J Pediatr.

[2] Gornitzky AL, Kim AE, O'Donnell JM, Swarup I (2020). Diagnosis and management of osteomyelitis in children: a critical analysis review. JBJS Rev.

[3] Lima AL, Oliveira PR, Carvalho VC, Cimerman S, Savio E (2014). Recommendations for the treatment of osteomyelitis. Braz J Infect Dis.

[4] Pinder R, Barlow G (2016). Osteomyelitis of the hand. J Hand Surg Eur Vol.

[5] Honda H, McDonald JR (2009). Current recommendations in the management of osteomyelitis of the hand and wrist. J Hand Surg Am.

[6] Pasquet J, Chevalier Y, Couval E, Bouvier D, Bolzinger MA (2015). Zinc oxide as a new antimicrobial preservative of topical products: interactions with common formulation ingredients. Int J Pharm.

[7] Merritt K (1988). Factors increasing the risk of infection in patients with open fractures. J Trauma.

[8] Mathieu L, Mottier F, Bertani A, Danis J, Rongiéras F, Chauvin F (2014). Management of neglected open extremity fractures in low-resource settings: experience of the French Army medical service in Chad. Orthop Traumatol Surg Res.

[9] Eidelman M, Bialik V, Miller Y, Kassis I (2003). Plantar puncture wounds in children: analysis of 80 hospitalized patients and late sequelae. Isr Med Assoc J.

[10] Imoisili MA, Bonwit AM, Bulas DI (2004). Toothpick puncture injuries of the foot in children. Pediatr Infect Dis J.

[11] Lew DP, Waldvogel FA (2004). Osteomyelitis. Lancet.

[12] Nandavar AV, Toledano T, Marino C, Khanna S, Sitnitskaya Y (2021). Contiguous osteomyelitis of distal extremities in children. Glob Pediatr Health.

[13] Howard CB, Einhorn M, Dagan R, Yagupski P, Porat S (1994). Fine-needle bone biopsy to diagnose osteomyelitis. J Bone Joint Surg Br.

[14] Jacobson IV, Sieling WL (1987). Microbiology of secondary osteomyelitis. Value of bone biopsy. S Afr Med J.

[15] Taj-Aldeen SJ, Rammaert B, Gamaletsou M (2015). Osteoarticular infections caused by non-Aspergillus filamentous fungi in adult and pediatric patients: a systematic review. Medicine (Baltimore).

[16] Osmon DR, Berbari EF, Berendt AR (2013). Executive summary: diagnosis and management of prosthetic joint infection: clinical practice guidelines by the Infectious Diseases Society of America. Clin Infect Dis.

[17] Kavanagh N, Ryan EJ, Widaa A (2018). Staphylococcal osteomyelitis: disease progression, treatment challenges, and future directions. Clin Microbiol Rev.

[18] Eckardt JJ, Wirganowicz PZ, Mar T (1994). An aggressive surgical approach to the management of chronic osteomyelitis. Clin Orthop Relat Res.

[19] Reese JH, Barrio J (1995). Surgical approaches to the management of osteomyelitis. Diagnosis and Management of Bone Infections.

